# Celebrating 20 Years of the *Journal of Insect Science* and Introducing More Equitable Peer Review

**DOI:** 10.1093/jisesa/ieaa141

**Published:** 2021-01-04

**Authors:** Phyllis G Weintraub

**Affiliations:** Department of Entomology, Agricultural Research Organization, Gilat Research Center, D.N. Negev, Israel

The *Journal of Insect Science* (*JIS*) was started 20 yr ago by Professor Henry Hagedorn as the first broad-based Open Access (OA) entomology journal (Weintraub 2015). His vision was to have a quality journal that was open to authors from all regions of the world and where the readers were not held hostage to market forces to receive information. In an effort to be more inclusive, when I started as editor-in-chief, I instituted three changes: 1) *JIS* abstracts could be published in a discoverable format in the author’s native language in addition to English, 2) readers could comment on published articles to stimulate and involve the general research community, and 3) I welcomed the publication of negative results.

Unfortunately, 2020 was a year of great consequences on many levels, two major examples being: 1) coronavirus and the disruption of societies around the world, and 2) inter-racial strife. As governments attempted to mitigate the transmission of coronavirus by closures and social distancing, research—both in the laboratory and field—was severely curtailed or, in some cases, brought to a stand-still. More than 120 journals responded to an influx of manuscripts on coronavirus by expediting publication and making articles free to the public (Berenbaum 2020). Since the virus is not insect-transmitted, *JIS* was not similarly inundated with manuscripts, but published articles have always been free to readers. However, the issue of inter-racial and inter-gender biases continues in publishing and this is something that *JIS* can and will be addressing.

**Fig. 1.**
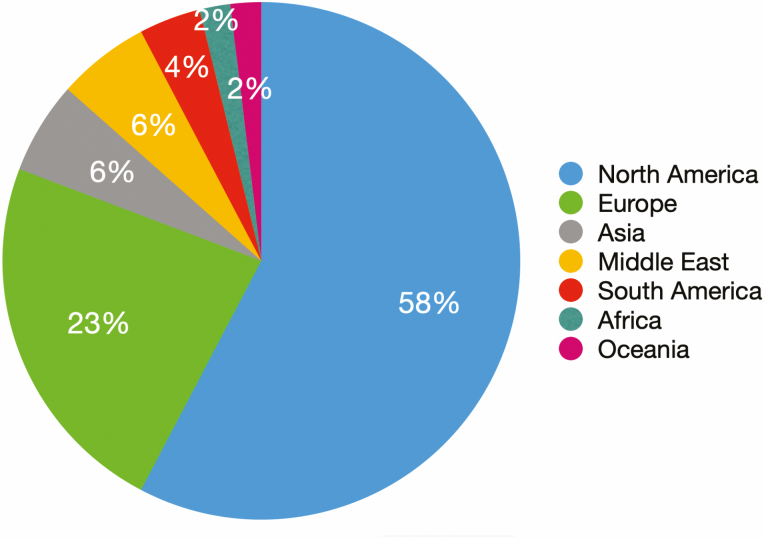
Distribution of subject editors for JIS.

In the 5 yr that I have been the Editor-in-Chief, the balance of women subject editors (SEs) has moved from deplorable to on par with the percent of women in the Entomological Society of America (~30%), of which *JIS* is one of the eight journals under its auspices. The *Journal of Insect Science* can boast of SEs from Africa, the Americas, Asia, Europe, and Oceania (Fig. 1). Lest I sound self-congratulatory, we at *JIS* can do better.

A cursory sketch of the history that brought an almost 300-yr-old method of peer-review (initiated in 1731 by the Royal Society of Edinburgh; Spier 2002) of scholarly publications to this point of change is as follows: until the 20th century, women were largely excluded from higher education; with the advent of World War II and the lack of men working in factories, women filled the void and were reluctant to relinquish their new-found independence and job satisfaction after the war ended; gradually, more and more women earned higher degrees in a wide variety of subjects and entered the science, technology, engineering and mathematics (STEM) workforce. The lack of parity with men and advancement in their fields lead to much research, eventually revealing the latent sexism in academia.

That the majority of publications from research examining lack of parity with white men focused on gender discrepancy, rather than racial, is due to the fact that women are fully 50% of the population, but a small fraction in STEM disciplines. An example of a study investigating gender bias and one of the most frequently cited (>700 to date) publications was by Moss-Racusin and colleagues (2012). In that study, identical CVs were assigned either an easily recognizable male or female name and rated by 127 science faculty from research-intensive universities. The anti-female bias in both male and female faculty was evident in ascribing significantly higher competency, hireability, probability of being mentored, and starting salaries for names recognizably male. This clearly represents an innate bias in universities and research institutes. Although less studied, similar biases exist for many minorities and authors whose names are not clearly recognizable or are clearly recognizable as ‘foreign’.

There are two potential changes to the predominate, long-established, single-blind peer-review system: open review and double-blind (also known as double-anonymous) peer review. The major drawback of an open review process, wherein authors know the identities of the reviewers and vice versa, is that early-career professionals would be reluctant to openly criticize established researchers for fear of retribution in often relatively small academic communities as direct criticism at meeting, poor reviews, or lack of promotion (Rodriguez-Bravo 2017).

Double-blind peer review means that, as with the typical single-blind review where the author doesn’t know who the reviewers are, additionally the reviewer does not know who the author(s) is/are as well, hence ‘double’. Beneficiaries of double-blind peer review are women (Budden et al. 2008), minorities (NSF 2017), and early career professionals who have yet to make a name for themselves (Tomkins et al. 2017).

As long as humans exist, there will be implicit biases so, instead of ignoring them or pretending they do not exist, we must try to rectify the situation, and as the editor-in-chief of a peer-reviewed journal, I strongly believe that double-blind review is the best method. ScholarOne allows for the electronic submission of blinded manuscripts, lacking the author names, institutions, and acknowledgments. However, double-blind review is not infallible:

1. There are some research areas within entomology which are so narrow, or have so few people researching the area, that an educated person might guess at the authorship.
2. Some researchers may be generally against double-blind reviews because they would lack the benefit of their established name. To circumvent this obstacle, some authors could try to reveal their identity by excessive self-citation, which *JIS* has always considered an unethical practice of plagiarism and rejected, or verbiage like ‘in our previous study’, which can be easily detected by the editor before being sent to reviewers.

I do not expect that the transition will perfectly smooth in the beginning. Since I review all manuscripts before they are sent to a subject editor, I anticipate some additional review time to look for excessive self-citation (which I have encountered in the past) and cryptic self-revealing verbiage. But, I am confident that whatever extra effort is needed will be rewarded in the long-run.

Moving to double-blind/double-anonymous peer review is an important shift as research has shown that gender balance is an important means of improving the quality of decision-making and ‘innovation dividends’ (Nielsen et al. 2017). I sincerely believe that the ultimate result of the change to a fair and balanced review system will be more women, early career, and non-white authors being published, thus advancing academically to higher positions.
